# Self-medication and knowledge among pregnant women attending primary healthcare services in Malang, Indonesia: a cross-sectional study

**DOI:** 10.1186/s12884-020-2736-2

**Published:** 2020-01-16

**Authors:** Rizka Novia Atmadani, Owen Nkoka, Sendi Lia Yunita, Yi-Hua Chen

**Affiliations:** 1 Pharmacy Department, Faculty of Health Science, University of Muhammadiyah Malang, Kampus II, Malang, Indonesia; 2 School of Public Health, College of Public Health, Taipei Medical University, Taipei, Taiwan

**Keywords:** Pregnancy, Over-the-counter medication, Knowledge, Healthcare service, Indonesia

## Abstract

**Background:**

Self-medication with over-the-counter (OTC) drugs is an important public health concern, especially in the vulnerable population of pregnant women due to potential risks to both the mother and fetus. Few studies have studied how factors, such as knowledge, affect self-medication. This study investigated self-medication and its associated factors among pregnant women attending healthcare services in Malang, Indonesia.

**Methods:**

A cross-sectional study was conducted from July to September 2018 in five healthcare services. A self-administered questionnaire was used and the data were analyzed using multiple regression models.

**Results:**

Of 333 female participants, 39 (11.7%) used OTC medication. Women with a higher level of knowledge of OTC medication were more likely to self-medicate—adjusted odds ratio (aOR) = 2.15, 95% confidence interval (CI) = 1.03–4.46. Compared with those with less knowledge, pregnant women with more correct knowledge of the possible risk of self-medication were less likely to self-medicate—aOR = 0.29; 95% CI = 0.14–0.60. The effect of a higher level of knowledge of OTC medication was significant among women who had middle school and lower education—aOR = 8.18; 95% CI = 1.70–39.35. The effect of correct knowledge on the possible risks of self-medication was significant only among women with high school and higher education—aOR = 0.17; 95% CI = 0.07–0.42.

**Conclusion:**

Imparting specific knowledge of the potential risks of using non-prescribed medication during pregnancy may help pregnant women navigate and more safely manage their OTC use. We also suggest further collecting data from more healthcare services, such as hospitals, to obtain more findings generalizable to the Indonesian community.

## Background

The use of medication during pregnancy is a public health concern. Globally, almost 50% of pregnant women use medication in the early weeks of gestation [1]. Using a web-based multinational study, Lupattelli et al. discovered that 81.2% of pregnant women used at least one type of medication, either prescribed or over-the-counter (OTC) [2]; over 65% self-medicated with OTC medication.

Self-medication, particularly with OTC medication, is considered a potential for harm for pregnant women [3–5]. The United States Food and Drug Administration’s (FDA’s) 1979 regulations categorized drugs by pregnancy risk. There are five categories, each marked by a letter: A, B, C, D, or X [6]. Only a few OTC medications or prescription drugs are of category A or B (indicating no evidence of risk to the fetus), whereas many drugs are of category C (indicating evidence of potential benefits outweighing potential fetal risks), or of categories D or X (indicating evidence of fetal risk) [7].

Indeed, medication use during pregnancy is a dilemma because the vulnerable population (i.e., pregnant women and children) is not included in clinical drug trials [8, 9]. Therefore, there is not enough data of the effects from such medicine on the vulnerable groups. One such study discovered an association between a pregnant woman’s use of aspirin and intracranial hemorrhage in her newborn baby [10]. Another study discovered an association between a pregnant woman’s use of valproic acid and the risk of neural tube defects in her fetus [11]. In general, studies on the fetal effects of self-medication are limited because of the complexity of the examination [12].

Despite the dilemma, prenatal self-medication is reportedly frequent. Studies on self-medication have reported its varying prevalence due to different study populations, design, and socio-cultural contexts. For instance, self-medication was reported among 12.5% of pregnant women in a study conducted in Netherlands [13], whereas a higher rate of 40% during pregnancy was reported by another study conducted in the United Arab Emirates [14]. In addition, there were inconsistent findings of the effect of different factors on self-medication during pregnancy from other studies [12, 15]. Studies concluded that factors such as one’s knowledge, beliefs, and socio-demographic background are associated with self-medication during pregnancy [16–21]. For instance, in studies conducted in Ethiopia and Italy, pregnant women with more knowledge of the risks of self-medication were less likely to self-medicate, compared with those with less knowledge [19, 22]. However, studies investigating about the knowledge of potential risk effects of those medications on the fetus are scarce.

In Indonesia, OTC medicines are readily available in drug stores, retail stores, or kiosks [23]. Previous studies have investigated self-medication in the Indonesian population, but they did not focus on prenatal usage [24]. Moreover, there is scant information in the literature on how a pregnant woman’s knowledge of OTC medication and her beliefs on the use of medication affects their practice of self-medication.

Due to self-medication’s potential for harm to both the mother and fetus, it is therefore imperative to study the prevalence of self-medication and factors associated with self-medication during pregnancy. In addition, such a study ought to focus on the factors of a pregnant woman’s knowledge of OTC medication and beliefs on the use of medication. Findings from such studies will help public health practitioners appraise the importance of a woman’s knowledge of the use of OTC medication. An appraisal will help in the formulation of health education programs to assist women in realizing how to safely manage their OTC use during pregnancy. For Indonesia in particular, although self-medication use for the general population has been investigated, the examination of the use during critical periods of women’s pregnancy has been lacking. Findings from those studies would be vital for tailor-made interventions to promote the safe use of medication during pregnancy for maternal and fetal health. Furthermore, it is important to examine effect modifiers between the relationship of knowledge of OTC medication with the practice of self-medication. Some examinations are helpful in the identification of high-risk groups with regard to self-medication during pregnancy.

Therefore, this study aims to examine (1) the proportion of pregnant women who self-medicated in this convenience sample in Malang, Indonesia; (2) the factors associated with the practice of self-medication during pregnancy; and (3) the moderating effects of socio-demographic characteristics on the relationship between knowledge of OTC medication and the practice of self-medication during pregnancy.

## Methods

### Study area

This study was conducted in Malang City and Malang Regency, Java, Indonesia. Malang Regency is the largest regency in East Java province. In 2017, its population was approximately 2,576,596 [25] and the population of Malang City was approximately 861,414 [26].

### Samples and data collection

This cross-sectional study was conducted from July to September 2018 at five primary healthcare services (*Puskesmas*, also called public health center) in Malang. Three healthcare services are located in the southeast area of Malang Regency, and two are located in the center of Malang City. A convenience sampling method was adopted. All pregnant women coming to the *Puskesmas* and queuing to see a healthcare provider (either a midwife or doctor) were eligible for inclusion in this study. Pregnant women who were unable to read or speak the language of Bahasa Indonesia were excluded from the survey as the data collection instrument was administered in this local language. Approximately 80 % of pregnant women agreed to participate in this survey among those who accessed care at that time. The questionnaires were self-administered in the waiting room at each of the healthcare service. They were collected on-site immediately after the questionnaires were completed. Upon completion, the interviewer checked the questionnaire and asked the respondent to review their responses if there were missing items.

The formula by Kish and Leslie (1965) was used for sample size calculation. With the use of previous data that 25% of pregnant women used OTC medication [27] and a 5% margin of error was expected, the sample size required for this study was 321 participants.

### Questionnaire development

A structured self-reported questionnaire was developed to assess pregnant women’s (1) health and pregnancy condition (pregnancy status, health condition, and health behavior), (2) knowledge of OTC medication during pregnancy, (3) beliefs about medication use during pregnancy, and (4) socio-demographic characteristics. The questionnaire was originally developed in English (Additional file 1) and translated into Bahasa Indonesia. For the evaluation of the content, semantics, and conceptual equivalence of the instruments in both the source and target languages, translation, back-translation, expert review and a pilot study was recommended by Guillemin et al. (1993) as guidelines for cross-cultural adaptation of health-related measures. The questionnaire utilized in this study was developed based upon these guidelines [28].

To translate the English instrument into Bahasa Indonesia version, we performed a forward and backward translation. First, a bilingual expert who was fluent in both English and Bahasa Indonesia translated the English version to Bahasa Indonesia. Another expert from a language center in Indonesia then back translated to English to ensure consistency of meaning. Then, two additional experts independently compared the original English instrument and the version translated back from Bahasa Indonesia to certify the equivalence and cultural relevance. An overall agreement was achieved. In addition, the instrument utilized was edited and modified based upon expert review. Four experts in pharmacy, public health, and epidemiology fields comprehensively reviewed the scope of this study and examined the content validity of the questionnaire in April, 2018. A pilot study was then conducted among 20 pregnant women [29] in May 2018 to assess practicability and face validity. This pilot study certified women’s understanding and feasibility of implementation. Minor modifications of the wording of the questions were further performed to ensure easier comprehension based upon experts’ evaluation.

### Outcome variable

The outcome measure was “self-medication” (specifically, of OTC medication) assessed by asking whether the pregnant women had used at least one type of OTC medication in their current pregnancy. They answered either yes or no.

### Independent variable

A pregnant woman’s knowledge of OTC medication, the main independent factor of this study, was evaluated relative to items generated from a literature review, yielding a total of 12 knowledge statements that was validly used previously [22, 30]. In our study, content validity index (CVI) calculated from expert review was utilized to quantify content validity. Based upon expert opinions along with CVI values over 0.8, all 12 questions were retained. Questions were further edited based upon the experts’ opinions. The Cronbach’s alphas for the questions on knowledge of OTC medication during pregnancy were 0.88 and 0.85 in the pilot study and in the final enrolled sample, respectively, indicating appropriate internal consistency.

Assessments of pregnant women’s knowledge of OTC medication involved statements such as “There are possible risks from the use of OTC medication during pregnancy” and “There is a need to consult a healthcare provider before taking OTC medication.” Each statement was accompanied by three possible responses: “yes,” “no,” and “do not know.” Items answered correctly were coded as “1” and items answered incorrectly (including those having the response “do not know”) are coded as “0.” These were summed into a knowledge score. As there were 12 statements, the knowledge scores ranged from 0 to 12. We used these total knowledge scores to estimate the change in the likelihood of self-medication per unit of change in knowledge.

In addition, we investigated whether women having knowledge above a certain level behaved differently in terms of OTC medication. We thus used the third quartile as a cut-off point to categorize knowledge scores into two (“high level of knowledge” and “low level of knowledge”) subcategories [31]. We also separately analyzed the two important questions/statements in the knowledge section of “Knowledge about the need to consult any healthcare provider” and “Knowledge about possible risk from taking OTC medication during pregnancy” to emphasize on the crucial and specific medication understanding of consultation with healthcare provider and possible risks during pregnancy.

### Other covariates

A pregnant woman’s beliefs regarding medication during pregnancy was measured using nine questions (six for medication and three for natural remedies usage) adopted from previously validated surveys in Norway [32], Saudi Arabia [33] and Belgium [34]. In our study, all nine questions were retained based upon experts’ evaluation and CVI values larger than 0.8, with minor editing performed corresponding with experts’ opinions. The Cronbach’s alphas were 0.82 and 0.7 in the pilot study and in the final enrolled sample, respectively, to indicate acceptable internal consistency. For the assessment of woman’s beliefs regarding medication during pregnancy in the first six questions, each question had a five-point Likert scale ranging from “strongly disagree” to “strongly agree.” The sum of the scores ranged from 6 to 30. This sum measured the level of a pregnant woman’s belief toward taking medication during pregnancy, with lower scores indicating a more positive belief. The first quartile was used as a cut-off point to categorize belief scores into two (“positive” and “negative”) subcategories.

Data on socio-demographic characteristics were also gathered. Two- and three-level variables were used. Two-level variables included gestational age (first vs. second and third trimesters), age (16–27 vs. 28–45 years), parity (0 vs. 1 or more children), education level (middle school and lower vs. high school and higher), number of antenatal care (ANC) visits (fewer than 4 vs. 4 or more), household income (fewer than 1.5 million Rupiah vs. 1.5 million Rupiah or more), and residence (urban vs. rural). Three-level variables included occupation (student, homemaker, and employed) and health behavior with regard to reading a drug’s accompanying leaflet (always, sometimes, and never).

### Statistical analysis

Data were entered and analyzed using SPSS version 18 (SPSS, Chicago, IL, USA). We used the chi-square tests and Fisher’s exact tests to analyze differences in socio-demographics (e.g., age, education), pregnancy related variables (e.g., number of ANC visits), health related variables (e.g., self-perceived heath status, checking drug leaflet), and knowledge on OTC medication in relation to self-medication. The variables that had been reported previously to potentially confound the association examined or were possibly related to main independent and outcome variables using simple logistic regression models (*p* ≤ 0.25) were considered for multivariable regression models selection [22, 35]. Logistic regression using the “enter method” with all potential covariates simultaneously included for consideration was performed for final model selection. All factors were reported with their crude and adjusted odds ratios (aORs) and their 95% confidence intervals (CIs). A *p* value of < 0.05 was considered statistically significant.

We also examined the interaction between knowledge and socio-demographic characteristics with the likelihood of self-medication. An interaction *p* value of < 0.1 [36] was used to indicate potential moderation effects and the warranting of further subgroup analyses.

### Ethical considerations

The Commission of Research Ethics of the University of Muhammadiyah Malang (E.5.a/226a/KEPK-UMM/VII/2018) provided ethical approval. Informed consent was sought from each respondent about the details of the study’s background, objectives, and providing information on the protection of the participant’s data. All respondents signed a written informed consent.

## Results

### Socio-demographic characteristics

In total, 340 respondents were enrolled for participation. After excluding those with missing or incomplete information on main variables, a valid sample of 333 women was included for analyses. Most participants were aged 16–27 years (54.4%), had attended high school or institutes of higher education (70.3%), had adequate ANC visits (68.5%), and were homemakers (72.1%) (Table 1).

**Table 1:** Distribution of participants by self-medication

Variables	Total (*n* = 333)	%	Self-medication		*p* value^a^
			Yes n (%)	No n (%)	
Self-medication in current pregnancy			39 (11.7)	294 (88.3)	
Gestational age (trimester)					0.470
First trimester	37	11.1	4 (10.3)	33(11.2)	
Second & Third trimester	296	88.9	35 (89.7)	261(88.8)	
Age					0.075
16–27	181	54.4	16 (41.0)	165 (56.1)	
28–45	152	45.6	23 (59.0)	129 (43.9)	
Parity					0.137
0	148	44.4	13 (33.3)	135 (45.9)	
1 or more children	185	55.6	26 (66.7)	159 (54.1)	
Education level					0.880
Middle school or lower	99	29.7	12 (30.8)	87 (29.6)	
High school or higher	234	70.3	27 (69.2)	207 (70.4)	
Number of ANC Visits					0.174
< 4	105	31.5	16 (41.0)	89 (30.3)	
≥ 4	228	68.5	23 (59.0)	205 (69.7)	
Occupation					0.901
Student	14	4.2	2 (5.1)	12 (4.1)	
Homemaker	240	72.1	27 (69.2)	213 (72.4)	
Employed	79	23.7	10 (25.6)	69 (23.5)	
Household Income					0.897
< 1.5 Million Rupiah	159	47.7	19 (48.7)	140 (47.6)	
1.5 Million Rupiah or more	174	52.3	20 (51.3)	154 (52.4)	
Residence					0.174
Urban	243	73.0	32 (82.1)	211 (71.8)	
Rural	90	27.0	7 (17.9)	83 (28.2)	
Self-perceived Health Status					0.997
Good	239	71.8	28 (71.8)	211 (71.8)	
Bad	94	28.2	11 (28.2)	83 (28.2)	
Checking drug leaflet					0.510
Always	184	55.3	19 (48.7)	165 (56.1)	
Sometimes	117	35.1	17 (43.6)	100 (34.0)	
Never	32	9.6	3 (7.7)	29 (9.9)	

^a^*p* value from chi square tests and Fisher’s exact test

### Self-medication during pregnancy

In total, 39 (11.7%) women self-medicated at least once during pregnancy. During pregnancy, the OTC medications used included antiemetic medicines (33%), cold and flu remedies (29%), anti-fever medication (15%), pain killers (13%), and others (10%). Among those who self-medicated during pregnancy, approximately 10.3% did so in their first trimester. No significant difference was observed in socio-demographic characteristics between those who self-medicated and those who did not (Table 1).

### Knowledge of OTC medication

Of the 12 statements measuring knowledge of OTC medication, 6 were answered correctly by over 60% of participants. The statement having the highest proportion (86.2%) of correct responses is “You need to consult with healthcare provider before or when taking OTC medication during pregnancy,” and the statement having the lowest proportion (28.8%) of correct responses is “Antibiotics is one of OTC medication” (Table 2). Table 2 lists the proportion of different knowledge responses segmented by self-medication. Women who took at least one OTC medication during pregnancy were more likely to correctly answer the statements “Vitamin is one of OTC medication” (79.5%) and “OTC medication can be in the dosage form of oral medication” (92.3%). By contrast, these women were more likely to incorrectly answer the statement “While taking OTC medication there is possible risk that OTC drugs can affect the baby” (59.0%).

**Table 2:** Knowledge of OTC medication

Statements	Total (*n* = 333) n (%)	Self-medication		
		Yes n (%)	No n (%)	*p* value^a^
OTC medications are primarily used to treat condition that do not need direct supervision from doctors				0.471
Correct	187 (56.2)	24 (61.5)	163 (55.4)	
Incorrect	146 (43.8)	15 (38.5)	131 (44.6)	
OTC medication is used for treating minor illness/minor injuries				0.057
Correct	229 (68.8)	32 (82.1)	197 (67.0)	
Incorrect	104 (31.2)	7 (17.9)	97 (33.0)	
Antibiotic is one of OTC medication				0.509
Correct	96 (28.8)	13 (33.3)	83 (28.2)	
Incorrect	237 (71.2)	26 (66.7)	211 (71.8)	
Vitamin is one of OTC medication				**0.003**
Correct	191(57.4)	31 (79.5)	160 (54.4)	
Incorrect	142 (42.6)	8 (20.5)	134 (45.6)	
The decision for using OTC medication is primarily made by consumers				0.162
Correct	205 (61.6)	28 (71.8)	177 (60.2)	
Incorrect	128 (38.4)	11 (28.2)	117 (39.8)	
You can buy OTC medication without a prescription from a doctor				0.333
Correct	234 (70.3)	30 (76.9)	204 (69.4)	
Incorrect	99 (29.7)	9 (23.1)	90 (30.6)	
You can buy OTC medication only in a Pharmacy				0.900
Correct	114 (34.2)	13 (33.3)	101 (34.4)	
Incorrect	219 (65.8)	26 (66.7)	193 (65.6)	
You need to consult with healthcare provider before or when taking OTC medication during pregnancy				0.094
Correct	287 (86.2)	37 (94.9)	250 (85)	
Incorrect	46 (13.8)	2 (5.1)	44 (15)	
The most dangerous time during pregnancy for consuming OTC medication is the first trimester				0.408
Correct	193 (58.0)	25 (64.1)	168 (57.1)	
Incorrect	140 (42.0)	14 (35.9)	126 (42.9)	
While taking OTC medication there is possible risk that OTC drugs can affect the baby				**0.002**
Correct	211 (63.4)	16 (41.0)	195 (66.3)	
Incorrect	122 (36.6)	23 (59.0)	99 (33.7)	
OTC medication can be in the dosage form of oral medication				**< 0.001**
Correct	215 (64.6)	36 (92.3)	179 (60.9)	
Incorrect	118 (35.4)	3 (7.7)	115 (39.1)	
OTC medication can be in the dosage form of topical medication				0.057
Correct	143 (42.9)	19 (48.7)	124 (42.2)	
Incorrect	190 (57.1)	20 (51.3)	170 (57.8)	
Knowledge total score (Mean;SD)	7.0 (3.1)	7.8 (2.6)	6.8 (3.2)	0.070^b^
Overall knowledge in binary outcome				0.087
Lower	212 (63.7)	20 (51.3)	192 (65.3)	
Higher	121 (36.3)	19 (48.7)	102 (34.7)	

^a^*p* value from chi square tests

^b^*p* value from student’s t-test

Bold *p* value means significant (i.e., *p* < 0.05)

### Beliefs about taking medication during pregnancy

Respondents’ beliefs about taking medication during pregnancy are presented in Table 3. They generally expressed negative belief toward medication use during pregnancy. A majority of respondents agreed with the following statements. “Pregnant women have a higher threshold for using medicine when pregnant than when not pregnant.” (84.1%). “It is better for the fetus that pregnant women refrain from using medicines during pregnancy, even when they were not pregnant and have an illness, they would have taken medicines.” (61.3%). “It is better for the fetus if the mother takes medicines and get well than having untreated illness during pregnancy.” (63.1%). By contrast, 56.5% of the sampled women disagreed with the statement “All medicines can be harmful to the fetus.”

**Table 3:** Beliefs on taking medication during pregnancy

Beliefs on medication in pregnancy		Total (*n* = 333) n (%)
All medicines can be harmful to the fetus^a^		
Agree		84 (25.2)
Uncertain		61 (18.3)
Disagree		188 (56.5)
It is better for the fetus that pregnant women refrain from using medicines during pregnancy, even when they were not pregnant and have illness, they would have taken medicines^a^		
Agree		204 (61.3)
Uncertain		24 (7.2)
Disagree		105 (31.5)
Pregnant women have a higher threshold for using medicine when pregnant than when not pregnant^a^		
Agree		280 (84.1)
Uncertain		27 (8.1)
Disagree		26 (7.8)
Many unborn children are saved because the mother take medicines during pregnancy when they have illness^a^		
Agree		178 (53.5)
Uncertain		87 (26.1)
Disagree		68 (20.4)
It is better for the fetus if the mother take medicines and get well than having untreated illness during pregnancy^a^		
Agree		210 (63.1)
Uncertain		61 (18.3)
Disagree		62 (18.6)
Doctors prescribe too many medicines to pregnant women^a^		
Agree		73 (21.9)
Uncertain		40 (12)
Disagree		220 (66.1)
Natural remedies can generally be used by pregnant women		
Agree		205 (61.6)
Uncertain		67 (20.1)
Disagree		61 (18.3)
Pregnant women more likely to use natural remedies during pregnancy		
Agree		185 (55.6)
Uncertain		67 (20.1)
Disagree		81 (24.3)
Pregnant women should not use natural remedies without advices from any health care providers		
Agree		236 (70.9)
Uncertain		32 (9.6)
Disagree		65 (19.5)
Belief on taking medication during pregnancy (summary index from 6 items)	**Total** **(*n*** **= 333)**	**Percentage** **(%)** 78.4 21.6
Negative	261	
Positive	72	

^a^Statement selected into a 6-item summary index

### Factors associated with self-medication

Table 4 reports the results from the multiple logistic regression analysis. Model 1 displays the crude odds ratio. Models 2 to 4 display the effects of knowledge, including the total knowledge score, binary knowledge outcome, and binary outcome of the two aforementioned important pieces of knowledge on self-medication during pregnancy, after adjusting for socio-demographics. Specifically, Model 2 indicates that the total knowledge score is significantly associated with self-medication—adjusted odds ratio (aOR) = 1.16, 95% CI = 1.02–1.33. Including overall knowledge with a binary outcome in Model 3, we observe that women with a higher level of knowledge of OTC medication were more likely to self-medicate compared with women with lower knowledge—aOR = 2.15, 95% CI = 1.03–4.46. Results from Model 4 indicate that compared with those who had a lower level of knowledge about the need to consult a healthcare provider before taking OTC medication during pregnancy, pregnant women who had high levels of such knowledge were more likely to self-medicate—aOR = 5.07, 95% CI = 1.11–23.2. However, pregnant women who had high levels of knowledge about the possible risks of OTC medication in the fetus were significantly less likely to self-medicate—aOR = 0.29, 95% CI = 0.14–0.60. Additionally, age remains significant in all adjusted models. This indicates that older pregnant women (28–45 years) were significantly more likely to self-medicate—aOR = 2.14, 95% CI = 1.01–4.50 (Model 4).

**Table 4:** Multiple logistic regression analysis of knowledge and other factors associated with self-medication during pregnancy

Variable	Model 1^a^	Model 2^b^	Model 3^c^	Model 4^d^
	cOR (95% CI)	aOR (95% CI)	aOR (95% CI)	aOR (95% CI)
Knowledge of OTC medication (all statements)				
Knowledge total score	1.12 (0.99–1.26)	1.16 (1.02–1.33)*	–	–
Overall knowledge				
Lower	1.00	–	1.00	–
Higher	1.79 (0.91–3.50)	–	2.15 (1.03–4.46)*	–
Knowledge regarding OTC medication with important statements				
Knowledge about the need to consult any healthcare provider				
Incorrect	1.00	–	–	1.00
Correct	3.26 (0.76–14.00)	–	–	5.07 (1.11–23.2)*
Knowledge about possible risk from taking OTC medication during pregnancy				
Incorrect	1.00	–	–	1.00
Correct	0.35 (0.18–0.70)**	–	–	0.29 (0.14–0.60)**
Socio-demographic characteristic				
Age				
16–27	1.00	1.00	1.00	1.00
28–45	1.57 (0.80–3.07)	2.20 (1.05–4.57)*	2.18 (1.05–4.53)*	2.14 (1.01–4.50)*

*OTC* over-the-counter, *cOR* crude odds ratio, *aOR* adjusted odds ratio, *CI* confident interval

^a^Crude Model

^b^Model 2 included knowledge total scores and all adjusting variables of socio-demographic characteristics (age, gestational age, education, occupation, residence, and household income), self-perceived health status, and check drug’s leaflet

^c^Model 3 included overall knowledge (lower vs. higher) and all adjusting variables listed in Model 2

^d^Model 4 included two knowledge statements of “the need to consult any healthcare provider” and “possible risk from taking OTC medication during pregnancy” and all adjusting variables listed in Model 2

*p* value * < 0.05; ** < 0.01

Finally, as both knowledge and belief are important factors, we further estimate the effects of knowledge on self-medication, after considering the effects of belief. The results were fairly consistent. Specifically, knowledge of OTC medication remains significant—aOR = 2.14, 95% CI = 1.03–4.46 after controlling for belief and other covariates. Meanwhile, no association was observed between belief and self-medication.

### Subgroup analysis for the effects of knowledge on self-medication by socio-demographics

The interaction terms of binary knowledge with education and binary knowledge with occupation had significant effects on self-medication (both *p* < 0.1). Subgroup analyses were then performed. Specifically, the effect of a higher level of knowledge on self-medication was significant among women with middle school or lower education—aOR = 8.18, 95% CI = 1.70–39.35—but not among women with high school or higher education (Table 5). Furthermore, the effect of knowledge of the possible risks of taking OTC medication during pregnancy on self-medication was significant only among women with high school or higher education—aOR = 0.17, 95% CI = 0.07–0.42 (Table 5). The moderation effects of occupation on the association between knowledge of possible risks and self-medication were not significant.

**Table 5:** Subgroup analysis for effects of knowledge on self-medication by socio-demographics

Outcomes	Education		Occupation	
	Middle school and lower	High school and higher	Homemaker	Employed
	aOR (95% CI)	aOR (95% CI)	aOR (95% CI)	aOR (95% CI)
Higher level of knowledge ^a^	8.18 (1.70–39.35)**	1.23 (0.53–2.86)	3.02 (1.18–7.70)*	0.67 (0.15–3.04)
Correct knowledge of risks of taking OTC medication during pregnancy^b^	1.09 (0.28–4.20)	0.17 (0.07–0.42)***	–	–

*OTC* over-the-counter, *aOR* adjusted odds ratio, *CI* confident interval

*p* value * < 0.05; ** < 0.01; *** < 0.001

^a^Knowledge was categorized into two levels: higher and lower. Results were adjusted for socio-demographic variables (such as age, gestational age, self-rated health status, education, occupation, household income, and residence) excluding variables treated as the effect modifier

^b^Knowledge was categorized into two levels: correct and incorrect answer. Results were adjusted for socio-demographic variables (such as age, gestational age, occupation, self-rated health status, household income, and residence)

## Discussion

This study aimed to investigate first, the proportion of pregnant women self-medicated in this collected sample and factors associated with self-medication and second, the potential moderation effects of socio-demographic characteristics. This study focused on pregnant women attending primary healthcare services in Malang, Indonesia. A higher level of knowledge was associated with a higher likelihood of self-medication during pregnancy. However, if women had knowledge of the risks from OTC medication, they were less likely to self-medicate. The effects of a higher level of knowledge on higher self-medication were significant among women with middle school or lower education, whereas the correct knowledge of potential risk effects was associated with a lower likelihood of self-medication among women with high school or higher education.

The proportion of self-medication during pregnancy in our sample was observed to be low (11.7%). Our findings were similar to those of studies conducted in the Netherlands (12.5%) [13], Nigeria (22.3%) [37], and Saudi Arabia (13.2%) [33]. The proportion in this study is however lower than that (40%) observed by a study conducted in the United Arab Emirates [14]. A US study reported that self-medication is common [12]. It is likely that pregnant women in Malang have more knowledge of the risks of taking OTC medication during pregnancy. This is evident in the high proportion of correct response for the statement such as “While taking OTC medication there is possible risk that OTC drugs can affect the baby” (63.4%). These findings are consistent with those of a study conducted in Saudi Arabia: 60% of pregnant women were able to name some medications to be avoided during pregnancy. This indicates relatively high levels of knowledge of the risk of using medication during pregnancy [33]. A lower proportion of self-medication in this current study population may also be partially explained by the relatively healthier group to investigate. Pregnant women in Malang who live in rural areas could rely more on herbal or traditional remedies than modern medicine.

Women with high levels of knowledge of OTC medication in our study were more likely to self-medicate during pregnancy. Because they knew more about the OTC medication, these women may be more likely to manage self-medication responsibly. This result is consistent with those of studies conducted in China [38], Nigeria [18], and India [39]. To propose some possible reasons explaining this phenomenon, first, a higher level of knowledge from prior experiences of self-medication to manage ill symptoms may increase the chance or competence for later practice of self-medication during pregnancy. Second, the faster alleviation of symptoms may also be associated with the use of alternative medication [18]. However, our findings are inconsistent with those of a study conducted in Delta State, Nigeria [37]. Examining the specific use of non-steroidal anti-inflammatory drugs (NSAIDs) as their main dependent factor might explain this inconsistency [40].

Two important knowledge statements were separately examined. Consistent with results from a study conducted in Italy [22], pregnant women were observed to be more likely to consult a medical professional before taking OTC medication. Such behavior is healthy and allows medical professionals to impart sound information on the use of medication during pregnancy. Another important observation was of pregnant women being less likely to self-medicate if they knew there were possible risks of taking medication during pregnancy. Imparting specific and crucial information about OTC medication may be more effective to help pregnant women safely manage their practice towards OTC medication.

Previous studies have reported socio-demographic characteristics, such as one’s education [18, 41–43], occupation [22, 41–43], health status [44], and household income [41] to be important factors on the likelihood of self-medication. Similarly, we observed that older pregnant women were significantly more likely to self-medicate, after including other covariates in the logistic regression. Nevertheless, this current study did not observe the significant effects of other socio-demographic characteristics on self-medication. Instead, the moderating effects of socio-demographic characteristics on the association between knowledge and self-medication were observed. The effects of a higher level of knowledge on taking at least one type of OTC medication were particularly significant among pregnant women with middle school or lower education (*p* < 0.05). Highly educated pregnant women with high levels of knowledge of the risks of taking OTC medication during pregnancy were less likely to self-medicate.

Our findings have important implications. Imparting specific knowledge of the potential risks of using non-prescribed medication during pregnancy may help pregnant women more safely manage their OTC use. The significant effects of a higher level of knowledge on self-medication among women with lower income and education levels may indicate a level of their competence that is a strength upon which a provider could build. This study was conducted in primary healthcare services (*Puskesmas*), a very basic type of healthcare service in Indonesia. Here, knowledge and experiences using OTC medication can be easily shared and spread. Most patients also come from low to middle-income families, especially in the rural area that is Malang Regency. Expanding the role of healthcare providers together with the provision of evidence-based information in prenatal health education is crucial to promote pregnant women’s safe management of OTC medication.

Our study is the first to examine self-medication during pregnancy in Indonesia. We identified factors associated with self-medication in the Malang population. To identify vulnerable segments of pregnant women for possible unsafe use of self-medication, we further performed subgroup analyses to examine moderation effects. These women should be targeted in the design and implementation of future health programs.

There are some limitations to this study. First, this study used a convenient sample drawn from the population in Malang area. As the areas selected for investigation may not be representative, the study’s results may not be generalized to all pregnant women in Indonesia. Second, the proportion of self-medication in this sample may have been underestimated. This study included women in all trimesters when administering the questionnaire. Thus, the subsequent medication use among women in their early trimester was not recorded in this study. Third, the study’s cross-sectional design inhibited causal inference.

## Conclusion

This study observed 11.7% of women in this convenience sample self-medicated during pregnancy. Knowledge and age were observed to be associated with self-medication during pregnancy. Our results demonstrated that knowledge of OTC medication in general, and knowledge of the possible risks of taking OTC medication during pregnancy in particular, were strongly associated with the use of self-medication among pregnant women in Malang.

This study can be improved by future studies using either larger cohorts or a case-control method to examine the effects of self-medication on the mother and child’s health during pregnancy and postpartum. Based on our findings, we also suggest collecting more data from more healthcare services, such as hospitals, to obtain more findings generalizable to the Indonesian community.

## Supplementary information

**Additional file 1.** Questionnaire – Self-medication Questionnaire.

## Data Availability

The data used/or analyzed during the current study is available from the corresponding author on a reasonable request.

## Abbreviations

ANC: Antenatal care
aOR: adjusted odd ratio
CI: Confidence interval
FDA: Food and Drug Administration
NTD: Neural tube defects
OTC: Over-the-counter
Puskesmas: Pusat kesehatan masyarakat
SPSS: Statistical Packages for Social Sciences
