# Supplementary material for: Self-medication and knowledge among pregnant women attending primary healthcare services in Malang, Indonesia: a cross-sectional study
Source: BMC Pregnancy Childbirth. 2020 Jan 16;20:42. doi: 10.1186/s12884-020-2736-2 (PMC6966862; doi:10.1186/s12884-020-2736-2)
Supplement: Supplementary file 1 — Additional file 1. Questionnaire – Self-medication Questionnaire. [file 12884_2020_2736_MOESM1_ESM.doc]

**Self Medication Questionnaire**

| Section 1 | A1 What is your gestational age now?  ◻ < 13 weeks  ◻ 13 ~ < 26 weeks  ◻ 26 ~ < 36 weeks  ◻ 36 weeks or more  A2 How many times you have visited Antenatal Care Services during your current pregnancy?  ◻ ………………..(Number of times)  ◻ Don’t know  A3 In general, how would you rate your health today?  ◻ very good  ◻ good  ◻ moderate  ◻ bad  ◻ very bad  A4 During your current pregnancy, did you ever use at least one over-the-counter (OTC) medication/drug ?  Note : **Over-the-counter** (**OTC**) drugs are medicines sold directly to a consumer without a prescription from a healthcare professional  ◻ Yes  ◻ No ***(If no skip to A6)***  A5 **If yes to A4**, specify the name of the OTC drug that you are using during pregnancy  **(You can have multiple answers)**  YES NO  Antiemetic  Any pain killer  Any cold and flu remedies  Anti-fever  Other  ***If Yes, please specify ....................***    A6 Do you normally read/check the accompanying leaflet content?  ◻ Always  ◻ Sometimes  ◻ Never |
| --- | --- |
| Section 2 | Note : Please tick one answer that you feel is the best answer for each statement.   |  | Statements | Yes | No | Don’t know | | --- | --- | --- | --- | --- | | B1 | OTC medications are primarily used to treat condition that do not need direct supervision from doctors |  |  |  | | B2 | OTC medication is used for treating minor illness/minor injuries |  |  |  | | B3 | Antibiotics is one of OTC medication |  |  |  | | B4 | Vitamin is one of OTC medication |  |  |  | | B5 | The decision for using OTC medication is primarily made by consumers |  |  |  | | B6 | You can buy OTC medication without a prescription from a doctor |  |  |  | | B7 | You can buy OTC medication only in a Pharmacy |  |  |  | | B8 | You need to consult with healthcare provider before or when taking OTC medication during pregnancy |  |  |  | | B9 | The most dangerous time during pregnancy for consuming OTC medication is the first trimester |  |  |  | | B10 | While taking OTC medication there is possible risk that OTC drugs can affect the baby |  |  |  | | B11 | OTC medication can be in the dosage form of oral medication |  |  |  | | B12 | OTC medication can be in the dosage form of topical medication |  |  |  | |
| Section 3 | Note : Please tick one answer that you feel is the best answer for each statement.   |  | Statements | Strongly disagree | Disagree | Uncertain | Agree | Strongly Agree | | --- | --- | --- | --- | --- | --- | --- | | C2-1 | All medicines can be harmful to the fetus |  |  |  |  |  | | C2-2 | It is better for the fetus that pregnant women refrain from using medicines during pregnancy, even when they were not pregnant and have illness, they would have taken medicines |  |  |  |  |  | | C2-3 | Pregnant women have a higher threshold for using medicine when pregnant than when not pregnant |  |  |  |  |  | | C2-4 | Many unborn children are saved because the mother take medicines during pregnancy when they have illness |  |  |  |  |  | | C2-5 | It is better for the fetus if the mother take medicines and get well than having untreated illness during pregnancy |  |  |  |  |  | | C2-6 | Doctors prescribe too many medicines to pregnant women |  |  |  |  |  | | C2-7 | Natural remedies can generally be used by pregnant women |  |  |  |  |  | | C2-8 | Pregnant women more likely to use natural remedies during pregnancy |  |  |  |  |  | | C2-9 | Pregnant women should not use natural remedies without advices from any health care providers |  |  |  |  |  | |
| Section 4 | D1 What is your age ? ..................................(years)  ◻ Don’t know  D2 How many living children do you have?  ◻ None  ◻ One  ◻ Two  ◻ Three or more  D3 What is the highest level of education you have completed?  ◻ Did not attend any school  ◻ Primary School  ◻ Middle School  ◻ High school  ◻ University or college or vocation  ◻ Other education; **please specify**: . . . . . . . . . . . . . . . . . . . . . . . .  D4 What was your job situation at the start of pregnancy?  ◻ Student  ◻ Homemaker  ◻ Health care professionals (physician, nurse, or pharmacist)  ◻ Employed in the non-healthcare sector  ◻ Other job; **please specify :............................................**  D5 What is your household’s income level per-month?  ◻ < Rp 1,5 Million  ◻ Rp 1,5 ~ < 3 Million  ◻ Rp 3 ~ < 8 Million  ◻ Rp 8 Million or more  D6 Where is the location of your house or residence?  ◻ Urban area  ◻ Rural area |
